# Subjective Visual Vertical and Horizontal Abnormalities in a Patient with Lateral Medullary Syndrome-A Case Report 

**Published:** 2015-01

**Authors:** Amit Kumar Tyagi, Gaurav Ashish, Anjali Lepcha, Achamma Balraj

**Affiliations:** 1 *AVC Department, Christian Medical College,Vellore,India.*

**Keywords:** Cerebrovascular Accident (CVA), Caloric Tests, Lateral Medullary Syndrome, Vestibular Function Tests.

## Abstract

**Introduction:**

Evaluation of persistent vertigo in post infarct patients is very important as the management depends on whether the cause is purely of central origin or due to associated vestibular affliction**.**

**Case Report:**

A patient with left sided dorsolateral medullary syndrome and persistent vestibular symptoms was evaluated. Vestibular test battery showed abnormal smooth pursuit, bilateral hyperactive caloric responses, and abnormal dynamic subjective visual vertical and dynamic subjective visual horizontal tests.

**Conclusion:**

Dorsolateral medullary infarctions (Wallenberg’s syndrome) typically cause a central vestibular tonus imbalance in the roll plane with ipsilateral deviations of perceived vertical orientation. The SVV and SVH tests may have a role in localizing the pathology in a patient with lateral medullary syndrome.

## Introduction

Lateral medullary syndrome also referred to as Wallenberg's syndrome is marked by a constellation of clinical features including nystagmus, Horner’s syndrome, dysphagia, decreased pain and thermal sensation on the ipsilateral face and contra lateral body, and ipsilateral hemi paresis (1).

In such cases inhibitory function of VOR may be suppressed, thereby increasing the excitatory state of the vestibular nucleus, resulting in bilateral hyperactive caloric response. This mechanism explains bilateral hyperactive caloric response seen in such lesions (2). The subjective visual vertical and horizontal (SVV/SVH) assessments are a clinical tool which evaluates an individual’s verticality to various spatial axes. Spatial orientation in relation to gravity is important for the maintenance of upright posture, gait, and most motor activities.The vestibular, visual, interoceptive and somatosensory systems combinely influence the spatial orientation, which may be affected in infarct patients to various degrees (3).

Prolonged vestibular symptoms in a patient with lateral medullary syndrome can be caused by abnormalities in the otolith pathway, which can be assessed by SVV and SVH. This helps the treating clinician to better localize the pathology and aid in better rehabilitation and recovery (1 ,4).

## Case Report

A fifty five year old gentleman with no past history of diabetes mellitus or hypertension, presented a history of a Cerebrovascular event three years ago followed by complaints of rotatory vertigo for three years. Episodes lasted 5-10 minutes but there was no hearing loss, tinnitus or hyperacusis associated with the vertigo. This episodic vertigo had been more frequent in the recent past. It was not associated with any headache or preceding aura. His Cerebrovascular event was diagnosed as left-sided lateral medullary syndrome with left hemi paresis, left Horner syndrome and left upper motor neuron facial palsy. MRI suggested multiple lacunar infarcts in the left medullar region. Over time the patient had shown slow but progressive improvement in all neurological deficits.

At the time of presentation he had residual intermittent nasal regurgitation, dysphagia to solid food, and change in voice. He also had tingling sensation, paresthesia, and decreased touch sensation of the left half of his face and the right side of his body. He had no history of seizures. 

Neurological examination revealed decreased sensation on the left half of the face and the right side of the body. Cranial nerve examination revealed that the Vagus nerve (right vocal cord palsy) and the trigeminal nerve (decreased touch sensation with paresthesia over the left half of the face) were affected; furthermore, gag reflex was sluggish. Cerebellar signs were positive on the left side with ipsilateral tilt on Romberg’s test. He had a wide-based gait with difficulty in turning. There was no significant spontaneous nystagmus. Gaze nystagmus was present on the left. Dix–Hallpike revealed non fatigueable left-beating horizontal nystagmus with no latency in sitting as well as supine positions.

**Fig. 1:**
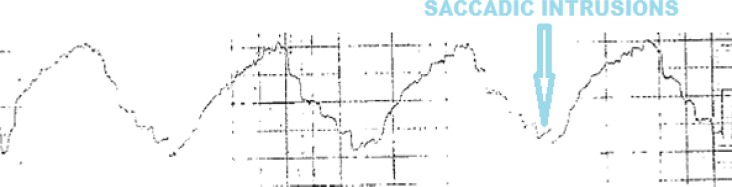
Saccadic intrusions of the smooth pursuit in Electronystagmography

**Fig. 2:**
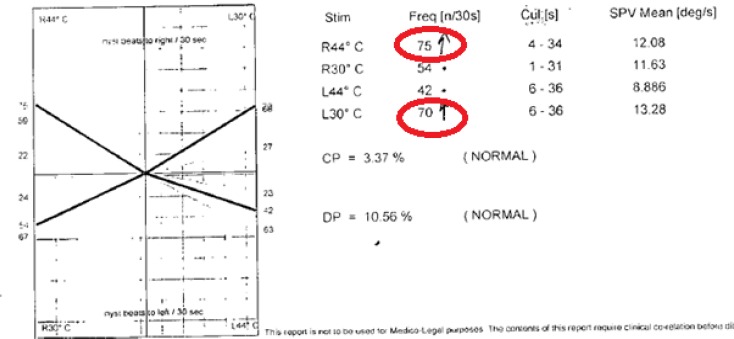
Bilateral hyperactive response in caloric test (Electronystagmography)

He had saccadic intrusions of the smooth pursuit evident in Electronystagmography (Fig.1). Optokinetic test was normal. Caloric tests revealed bilateral hyperactive response (Fig.2) pure tone audiometry and impedance audiogram was within normal limits.

Subjective visual vertical (SVV) and subjective visual horizontal (SVH) were tested using a pre-programmed computed equipment (MUS_VS-V1.3.2.RevB-Synapsis company). The subject was seated in a darkened room (to avoid any visual cues). Head and neck was stabilized in an erect neutral position using a headband fixed to the patient’s high back chair. The stimulus was projected on a large screen monitor mounted at a distance of 2 metres, in front of the patient.

The patient was fitted with a contour mask with binocular vision fitted with a set of 3 obturators so as to further reduce the chance of any visual cues. The stimulus was a projected vertical illuminated ‘line’ on the screen provided by the software from the SVV and SVH equipment**.** It was presented at a preset angle (between 5 degrees and 30 degrees) in a random fashion. He was required to adjust the ‘line’ to absolutely vertical and horizontal as perceived by him, using a joy stick (remote controlled potentiometer). For the dynamic assessment, the background was made to rotate clockwise or anti-clockwise. It was repeated six times, each for static and dynamic settings and values were recorded. The above procedure was in accordance with standard recommendations (5,6). 

Static SVV was 1.45 degree and static SVH was 1.81 degree, which was within normal values. The dynamic SVV and SVH were found to be 5.48 degrees and 3.08 degrees which were noticeably abnormal (6).

Imaging three years after a Cerebrovascular event revealed an old left PICA (posterior inferior cerebellar artery) infarction and MRA suggested absence of flow in the left vertebral artery. He was advised a short course of vestibular suppressants and prolonged vestibular rehabilitation exercise. He was significantly relieved of his symptoms after four months.

## Discussion

Wallenberg first reported lateral medullary syndrome in 189, which is now referred to as Wallenberg's syndrome.(7) It is a constellation of symptoms most commonly resulting from occlusion of the ipsilateral intracranial portion of vertebral artery, occasionally due to the compromise of the lone posterior inferior cerebellar artery (7,8), resulting in a zone of infarction involving the medial, superior, and/or inferior vestibular nuclei (9-11).

It is characterized by nystagmus, Horner’s syndrome, dysphagia, decreased pain and thermal sensation on the ipsilateral face and contra lateral body, and gait ataxia which are important in the differential diagnosis of peripheral vertigo (12). Cerebellar infarction can overlap substantially with benign conditions and is commonly misdiagnosed (around 35%) (13).

Case reports also describe unique clinical presentations of vascular vestibular syndrome characterized by severe vertigo, ipsilesional spontaneous nystagmus, and contralesional axial lateropulsion due to the involvement of medial PICA, and mimics peripheral vertigo (14,15).

Infarction of the medulla and inferior cerebellum can be due to involvement of PICA. Dysfunction of inferior vestibular nuclei in such cases causes a severe persistent vertigo and even vomiting (16). Although recent literature insist on the occlusion of the intracranial vertebral artery as the main cause of Wallenberg’s syndrome, only a fraction of these cases manifest due to sole involvement of PICA        (17).

PICA most often originates from the vertebral artery, and rarely from the basilar artery. The common trunk of the PICA divides into a medial branch and lateral branch. Inferior vermis including the nodulus and uvula, and the inferior cerebellar hemisphere are supplied by medial PICA leading to positive cerebellar signs in such cases (18). Cerebellar signs, particularly ataxia, and direction changing gaze-evoked nystagmus (71% and 54%, respectively) are usually seen in PICA infarcts; however these findings can also be found in cases with peripheral vertigo(19).

In normal individuals, the cerebellar flocculus inhibits vestibular nucleus neurons, hence inhibiting the VOR (20,21). Pathology in this region affects this inhibitory function, increasing the excitatory state of the vestibular nucleus, resulting in bilateral hyperactive caloric response. This mechanism explains bilateral hyperactive caloric response commonly seen in such lesions (2). However this response may be present without any apparent cause, such as in anxiety or to the intake of psychoactive drugs (2,22).

The subjective visual vertical and horizontal (SVV/SVH) assessment is a clinical tool that evaluates an individual’s capacity to determine whether an object is aligned in the vertical or horizontal position, without reference. Spatial orientation in relation to gravity is important for the maintenance of upright posture, gait, and most motor activities (23). The vestibular, visual, interoceptive and somatosensory systems combinely have a major role to play in spatial orientation. The test is administered by asking an individual to align a projected luminous bar on a screen to a position that the individual judges to be vertical by using a remote controlled potentiometer. 

The tilts of the lines are measured in degrees. The ability to judge whether the bar is aligned with the real vertical indicates the integrity of visual and vestibular otolithic information (23). Tilting of the SVH or SVV in a normal sighted subject seated upright indicates asymmetry of the resting neural activity of the vestibular system. This may be attributed to unilateral loss of peripheral vestibular function or by central nervous lesions. The magnitude of tilt of SVH usually corresponds to the degree of asymmetry of the resting neural activity (24).

Unilateral vestibular disturbance causes an imbalance in the resting neuronal activity and therefore the otolith organs of the contralateral ear “push” the SVV up to 20 degrees to the diseased side (25). 

On the other hand, lower brainstem lesions involving the vestibular nucleus can result in an SVV offset toward the side of lesion. On the contrary, upper brainstem lesion involving the interstitial nucleus of Cajal or cerebellar lesions results in an SVV that is offset away from the side of lesion (26). Thus, central lesions and peripheral vestibular (otolithic) lesions both can cause SVV or SVH abnormalities.

All unilateral brainstem lesion *caudal* to the upper pons cause *ipsilateral *Occular tilt (OT) with concurrent *ipsilateral* SVV tilts and all lesions rostral to this pontine level cause *contralateral* *tilts* of Occular Tilt and SVV. 

Pathological tilts of SVV and SVH are caused by dysfunction of the tonic bilateral vestibular inputs, which stabilize the eyes and head in a normal upright position in the roll plane and dominate our perception of verticality (26). 

Dynamic SVV and SVH test is a useful tool for the evaluation of such patients, especially in long standing symptoms when vertigo is often replaced by more nonspecific symptoms (instability, loss of balance, and oscillopsia) suggesting residual otolith damage.

In the acute phase, SVV generally shows such a large degree of error that it is easily picked up by static test. Thus the dynamic test-which is more sensitive, is better suited for revealing false negatives arising from the application of the static test alone. Therefore, this contributes to a better diagnosis and planning of treatment and rehabilitation (27).

## Conclusion

Prolonged vestibular symptoms in a patient with lateral medullary syndrome can be due to abnormalities in the otolith pathway. Bilateral hyperactive caloric response may be observed in central vestibular diseases. SVV and SVH are useful tests to detect these abnormalities and may have a role in localizing the pathology. Dynamic SVV and SVH abnormalities may be present long after static tests have reverted to normal. Hence dynamic SVV and SVH are important tests in the work up of a patient with vestibular symptoms following brainstem stroke. Proper counseling and rehabilitation measures can thus be offered to such patients. 
